# Anti-DNA virus agent cidofovir - loaded green synthesized cerium oxide nanoparticles (Nanoceria): Nucleic acids (DNA and RNA) binding affinity and cytotoxicity effects

**DOI:** 10.18632/oncotarget.28774

**Published:** 2025-11-06

**Authors:** Nahid Shahabadi, Saba Zendehcheshm, Fatemeh Khademi, Mohammad Mahdavi

**Affiliations:** ^1^Inorganic Chemistry Department, Faculty of Chemistry, Razi University, Kermanshah, Iran; ^2^Medical Biology Research Center, Health Technology Institute, Kermanshah University of Medical Sciences, Kermanshah, Iran

**Keywords:** CeO_2_ NPs, green synthesis, DNA interaction, RNA interaction, cytotoxicity

## Abstract

In this study, cerium oxide nanoparticles (CeO_2_ NPs) were synthesized using a green chemistry approach, utilizing quince fruit (Cydonia oblonga) peel extract as a non-toxic reducing and stabilizing agent. This environmentally friendly technique represents a novel approach to nanoparticle fabrication, emphasizing sustainability in nanotechnology. The surface of the green-synthesized CeO_2_ NPs was further functionalized with cidofovir (CDV), an anti-DNA virus agent, to develop a dual-functional therapeutic platform with potential anticancer and antiviral applications. The successful synthesis and modification of CDV-loaded CeO_2_ NPs (CDV- CeO_2_ NPs) were confirmed through a series of characterizations, including FT-IR, zeta potential, TEM, SEM-EDX, DLS, and UV-Vis analyses. The cytotoxic effects of CDV, CeO_2_ NPs, and CDV- CeO_2_ NPs were evaluated against the MCF-7 breast cancer cell line using the MTT assay, revealing that the loading of CDV onto CeO_2_ NPs significantly enhanced its anticancer efficacy. Furthermore, the interaction of CDV-CeO_2_ NPs with nucleic acids (DNA and RNA) was investigated through absorption and fluorescence studies, demonstrating a strong binding affinity and suggesting the potential of these nanoparticles as highly specific chemotherapeutic agents. The novelty of this work lies in the innovative green synthesis method, the dual-functional therapeutic application, and the enhanced biological activity of the CDV-CeO_2_ NPs, which collectively position these nanoparticles as promising candidates for future cancer and antiviral therapies.

## INTRODUCTION

**Figure 1:**
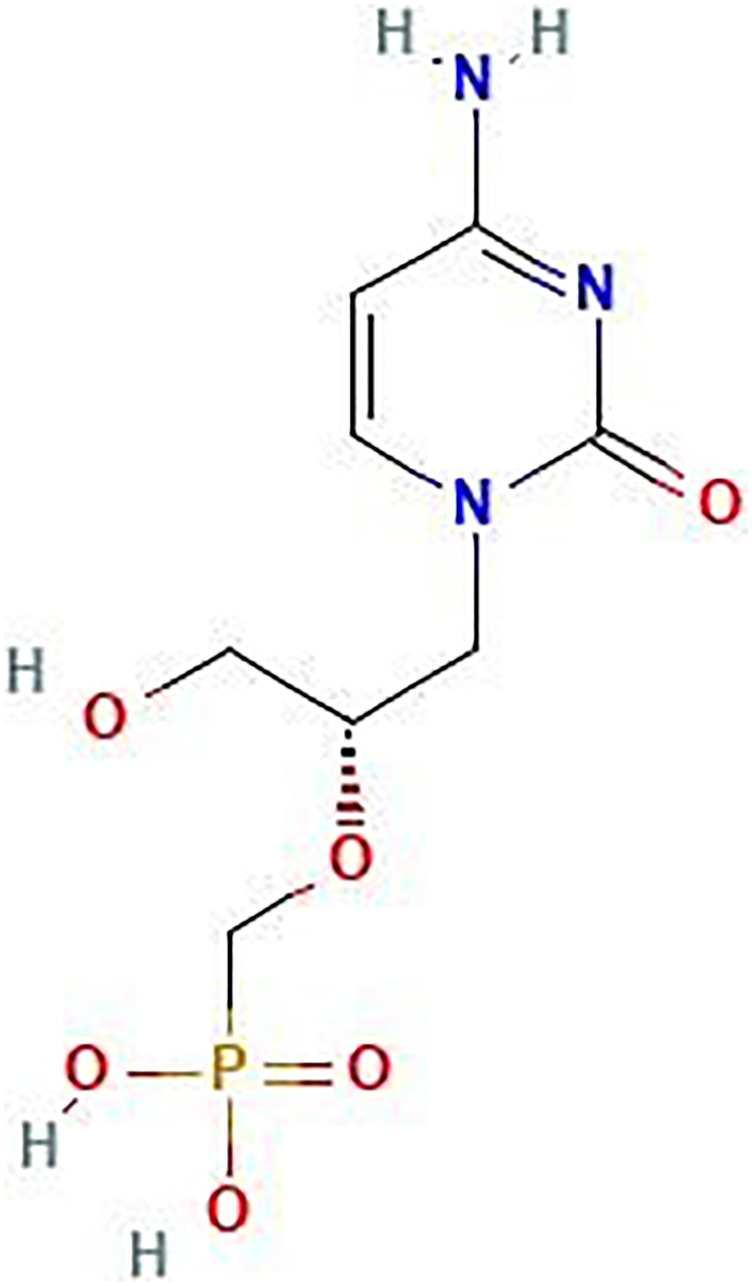
The molecular structure of antiviral drug cidofovir (CDV).

In recent years, metal oxide nanoparticles (MO-NPs) have attracted increasing attention due to their exceptional properties and wide-ranging applications. The production of MO-NPs has become a focal point in nanotechnology, as their demand continues to grow in various industries and medical fields, such as fillers, sterilizers, optical devices, catalytic products, drug delivery and antimicrobial agents [1]. Cerium, a rare earth element, exhibits unique chemical behavior that distinguishes it from transition elements, alkaline earth and post transition. This behavior can be attributed to the shielding effect and the characteristics of its 4f orbitals, which endow rare earth elements with specific catalytic, magnetic, photocatalytic, and electronic properties [2, 3]. These distinctive features enable innovative applications that cannot be achieved with transition elements. CeO_2_ NPs, or nanoceria, primarily exhibit two self-regenerating oxidation states: Ce^4+^ and Ce^3+^. Nanoceria possesses a range of remarkable properties, including such as nonstoichiometric, reduction behavior, oxygen storage capacity, large magnetic moment, high complexation property, and high conductivity. Furthermore, small-scale ceria can exhibit unique characteristics, such as a larger band gap compared to its bulk counterpart [4]. Nanoceria has shown promise as an effective treatment for neurodegenerative diseases and holds numerous potential applications as antioxidants in biological systems, catalysts, and in fuel cells. Moreover, other biological properties of nanoceria have been reported, such as anti-inflammatory, antitumor [5], and enzyme mimetic activities including scavenging hydroxyl radicals, superoxide dismutase (SOD), peroxidase and catalase have also been reported [6, 7]. Nanoceria has been the focus of many *in vivo* and *in vitro* researches, especially in the field of tumor microenvironments (TME) as a cytotoxic agent [8]. Its oxidative stress-induced properties have been found to increase tumor cell membrane leakage without harming healthy cells, which can aid in the design of new drugs [9, 10]. Moreover, nanoceria has been employed to protect primary cells from radiation therapy’s adverse effects and serve as potential drug delivery platforms [11]. The scientific literature highlights the significance of nanoceria in the development of novel therapeutic drugs [12–16]. Various synthesis methods for nanoceria have been developed, such as sol-gel [17], hydrothermal [18], precipitation [19], solvothermal [20], emulsion technique [21], combustion synthesis [22], microwave approach [23], and thermal decomposition [24]. However, chemical methods involve toxic chemicals, capping agents or additives, and non-polar solvents, making them unsuitable for biomedical applications. As a result, many researchers are searching for biocompatible and eco-friendly methods to synthesize nanoparticles and are exploring biosynthesis techniques. In nanotechnology, the biosynthesis process plays a critical role as it eliminates harmful byproducts from specific chemical reactions and enables a solvent-free organic synthesis protocol. Additionally, it is easily scalable for large-scale production and is cost-effective. The extracts of plant, egg white, honey, and fungal extracellular compounds, have been employed as both reducing and capping agents in synthesis of nanoparticle [15, 25, 26]. These bio-components contribute to the production of nanocrystalline metal oxide nanoparticles with varying morphologies and sizes. The Quince Fruit (*Cydonia oblonga*) is a member of the Rosaceae family and is known by various names such as “Bahee Dana” in Urdu, “Beh” in Farsi, “Strythion” in Greek, and “Bihi” in Hindi. It is indigenous to western Asia, stretching from Iran to Turkestan, and has been used since ancient times, with its origin dating back to Persia around 4000 BC. The fruit has spread throughout the Mediterranean basin to the west and Afghanistan to the east, along with the flourishing civilizations of that era [27, 28]. Nowadays, quince is cultivated in various regions worldwide, including many European nations, Oceania, South America, Australia, and North and South Africa [29], the Mediterranean, and parts of Asia. It grows wildly in different regions of Iran, such as Azerbaijan, Gilan and Golestan. Quince fruit is composed of vitamins B1, B2, PP, and C, organic acids (malic acid, citric acid), carbohydrates (starch, fructose, glucose), carotene, tannins, aromatic compounds, fiber magnesium, phosphorus, potassium salts, calcium, proteins. Notably, it contains well-known antioxidants like caffeoylquinic acids and rutin, with the peel being a rich source of caffeoylquinic acid, along with other significant flavonoids such as quercetin 3-galactoside, kaempferol-3-rutinoside and kaempferol 3-glucoside [30]. These compounds contribute to the formation of nanosistines and alter their surface. The fruit’s most beneficial components are the polyphenols with antioxidant potential, found in its peel, leaves, and seeds [31–34]. The current approach to inhibit cancer cell growth using anticancer drugs like doxorubicin, paclitaxel, and cisplatin is associated with undesirable side effects such as fever, vomiting, mouth sores, and hair loss. Developing effective anticancer drugs with fewer side effects is a time-consuming process. However, drug efficacy can be improved, side effects can be reduced, or drugs can be delivered in a targeted manner to expedite the process [35]. Antiviral drugs are a specific class of medications used to treat viral infections [36]. CDV (see Figure 1), also known as (S)-1-(3-hydroxy-2-(phosphonomethoxy) propyl) cytosine dihydrate (HPMPC), is an antiviral medication that belongs to a novel family of drugs. Cervical cancer and a significant proportion of head and neck squamous cell carcinomas (HNSCC) are caused by the human papillomavirus (HPV). Cidofovir has been found to be effective in treating various HPV-induced benign and malignant hyperproliferation. Mertens et al. reported that the antiproliferative effects of CDV are due to its incorporation into DNA, which leads to DNA damage [37]. Hadaczek and colleagues reported that CDV exhibits potent antineoplastic activity against HCMV-infected glioblastoma cells by inhibiting HCMV gene expression and inducing cellular apoptosis [38]. They also found that CDV induces glioblastoma cell death in the absence of HCMV infection by integrating into tumor cell DNA and promoting double-stranded DNA breaks, leading to apoptosis. When combined with ionizing radiotherapy, the standard treatment for glioblastoma in humans, CDV enhances radiation-induced DNA damage and further induces tumor cell death. The combination of CDV and radiotherapy significantly prolonged the survival of mice with intracranial glioblastoma tumors. Investigating the interactions between small molecules and nucleic acids has been a significant area of research for many years [39, 40], RNA and DNA are important pharmacological targets for many antibiotics and anticancer drugs. Comprehensive knowledge of the molecular mechanisms underlying the interaction between small ligands and nucleic acids is essential for the development of potent RNA-binding antiviral compounds and DNA- or RNA-based anticancer drugs. This study presents the first-ever production of CDV-loaded green synthesized cerium oxide nanoparticles (CDV-CeO_2_ NPs) derived from quince fruit peel extract and their characterization studies. We have investigated the structural and anticancer properties and the interactions between DNA/RNA and CDV-CeO_2_ NPs. This study is the first of its kind on the green synthesis of CDV-CeO_2_ NPs employing quince peel extract and their nucleic acid interaction investigations. Although some nanocarriers have been developed and utilized for targeted anticancer drug delivery, their effects on processes such as DNA and RNA interactions are not fully understood. Therefore, this study aimed to explore the DNA and RNA interactions of CDV-CeO_2_ NPs and demonstrate the impact of CDV-CeO_2_ NPs on binding mode employing physico-chemical techniques.

## RESULTS AND DISCUSSION

### The characterization of CeO_2_ NPs synthesized through a green process

#### Electronic absorption titration

**Figure 2:**
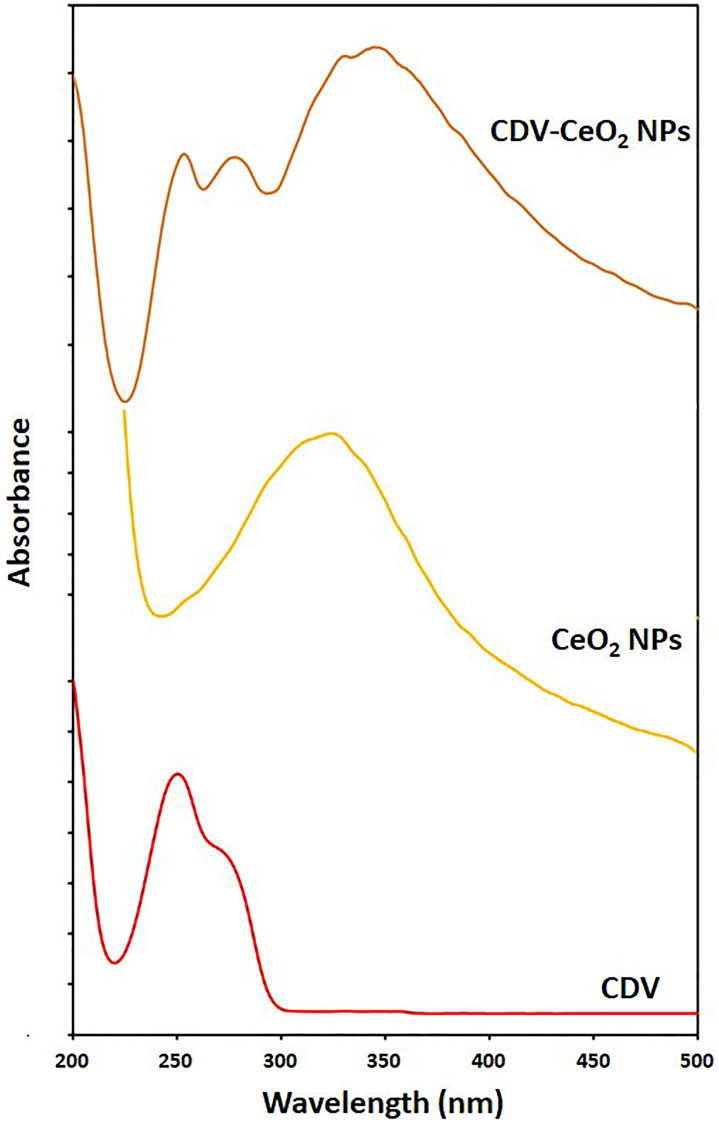
UV–vis spectra of CDV, CeO_2_ NPs and CDV-CeO_2_ NPs.

Figure 2 depicts the UV-Visible spectra of green synthesized CeO_2_ NPs, CDV, and CDV-CeO_2_ NPs. The absorption peaks are between 250 to 400 nm for green synthesized CeO_2_ NPs which, is due to the charge transfer from O_2P_ to Ce_4f_ orbitals [41]. As can be seen, the absorption peak between 250 to 400 nm for CDV-CeO_2_ NPs shifted toward long wavelength about 21 nm, which authenticated that the loading of drug had successfully happened. Moreover, the bands at 253 nm and 278 nm were developed (similar to the maximum wavelength of the drug), which proved the presence of surface loaded on the structure of the CeO_2_ NPs [42].

To determine the drug loading amount on the CeO_2_ NPs that were synthesized using green methods, an ultraviolet-visible spectrophotometer was used. The supernatant, collected through centrifugation, was used to measure the amount of unloaded drug at 249 nm. The efficiency of the drug loading was then determined using the standard curve equation.


y=34.288x+0.1379 (1)

The efficiency of CDV loading was estimated about 30%.

#### Zeta Potential and DLS measurements of CeO_2_ NPs

**Figure 3:**
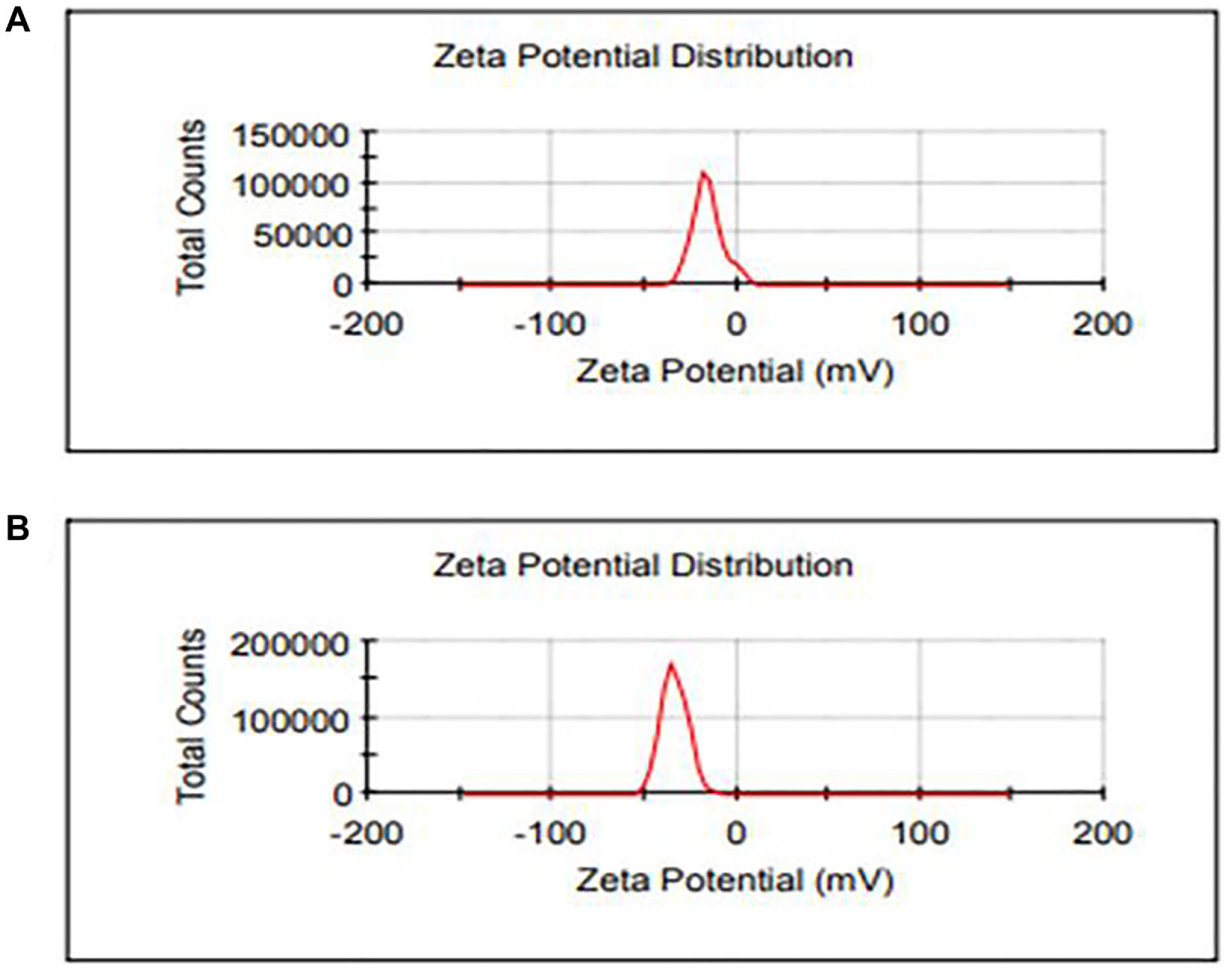
Zeta potential distribution of (**A**) CeO_2_ NPs and (**B**) CDV-CeO_2_ NPs.

**Figure 4:**
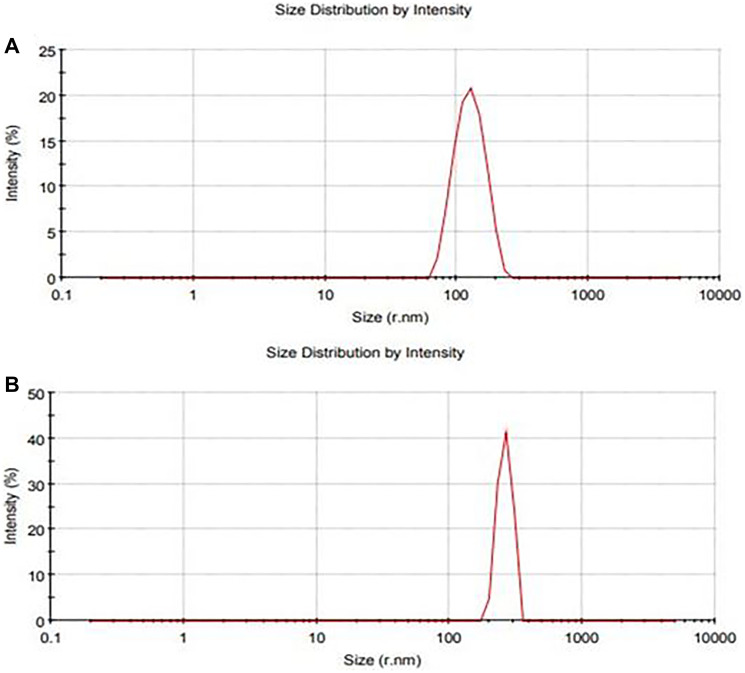
DLS particle size analysis of (**A**) CeO_2_ NPs and (**B**) CDV-CeO_2_ NPs.

DLS was utilized to measure the size distribution of CeO_2_ NPs and CDV-CeO_2_ NPs. Zeta sizer was also employed to identify the surface charge on CeO_2_ NPs and CDV-CeO_2_ NPs. Figure 3 represents the negative surface charge value (−15.6 and −33.5 mV) on the CeO_2_ NPs and CDV-CeO_2_ NPs, respectively, which indicates the negative-negative repulsion that leads to their high degree of stability [43]. Monodispersity of CeO_2_ NPs and CDV-CeO_2_ NPs were also reflected by the sharpness of the DLS peaks [44]. As revealed in Figure 4 the average size of CeO_2_ NPs and CDV-CeO_2_ NPs is 128.4 and 261.2 nm, respectively. It is clear that the CDV-CeO_2_ NPs’ average size increased, confirms the presence of a layer of CDV on the CeO_2_ NPs [45].

#### FE-SEM and EDX analysis

**Figure 5:**
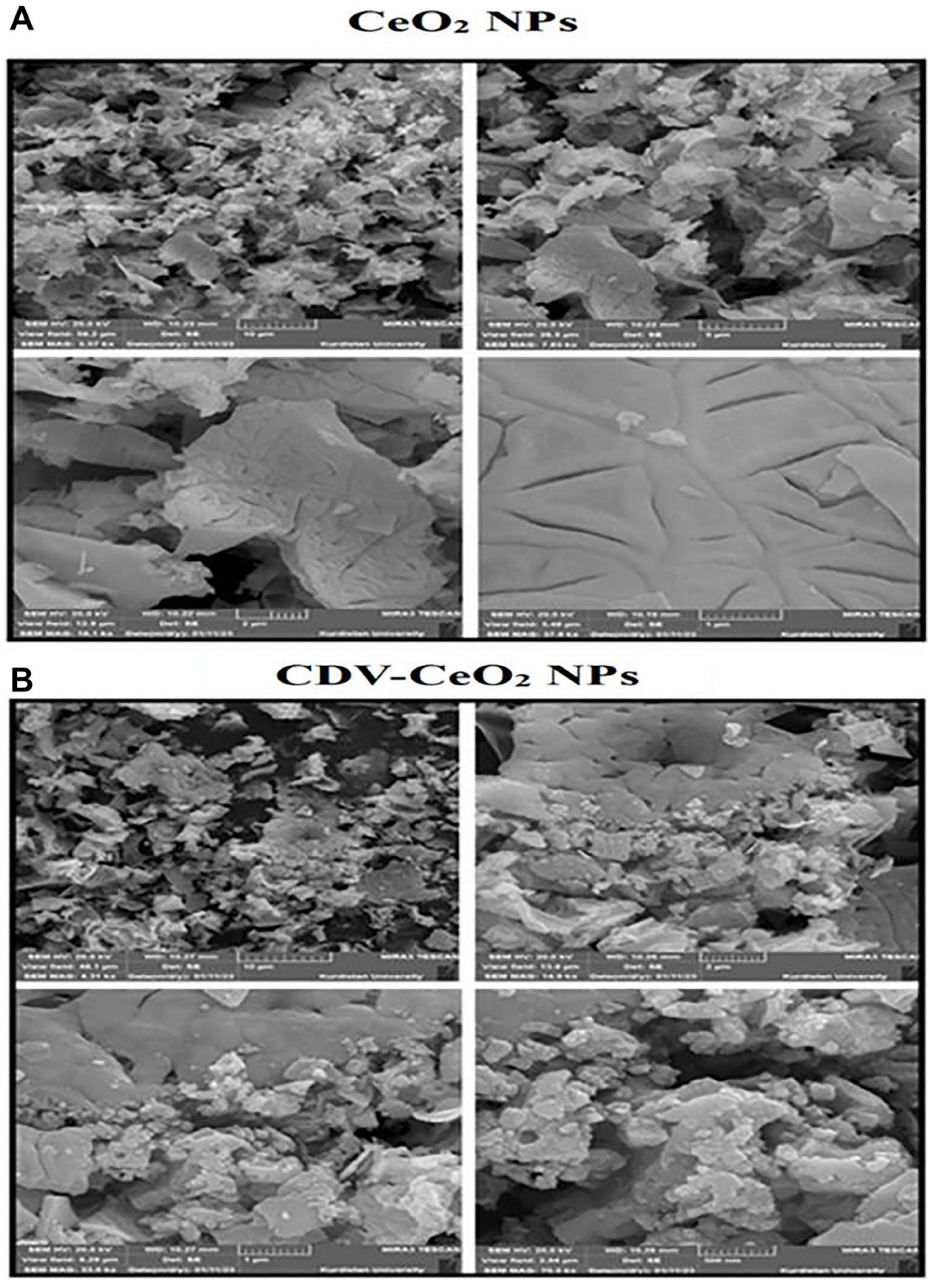
SEM images of (**A**) CeO_2_ NPs and (**B**) CDV-CeO_2_ NPs.

**Figure 6:**
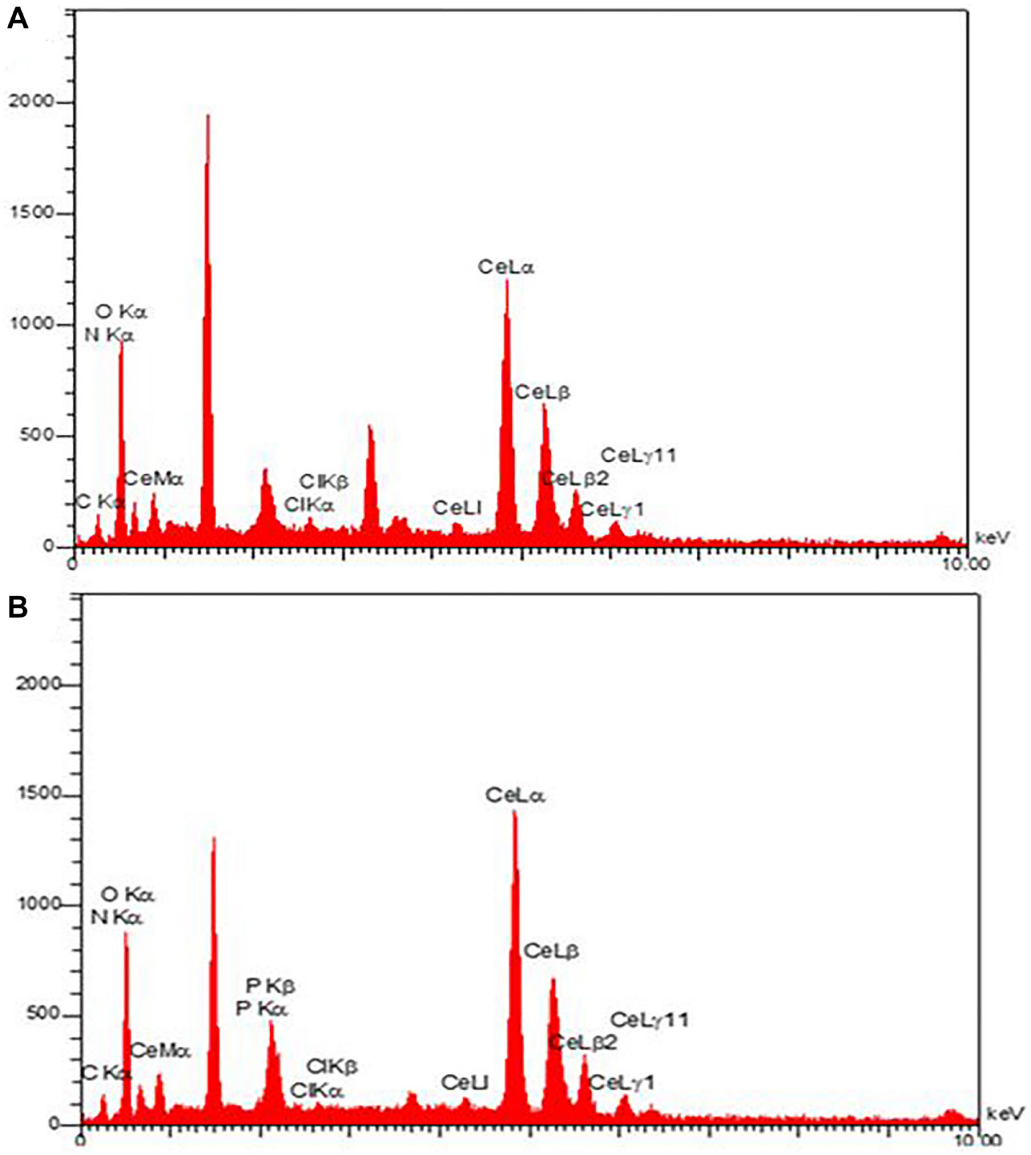
EDX images of (**A**) CeO_2_ NPs and (**B**) CDV-CeO_2_ NPs.

FE-SEM technique was utilized to analyze the morphology of CeO_2_ NPs and CDV-CeO_2_ NPs. FE-SEM images demonstrated agglomeration of CeO_2_ NPs. The CeO_2_ nanoparticles exhibit a flake-like structure (see Figure 5A) [46]. As can be seen, the CDV loading on the CeO_2_ NPs structure leads to an alteration in the CeO_2_ NPs’ morphology (see Figure 5B). It is obvious that the small granules or grains confirm the presence of the drug on CeO_2_ NPs [47]. The compositional analysis of CeO_2_ NPs and CDV-CeO_2_ NPs were illustrated by utilizing EDX analysis (see Figure 6). EDX analysis results of CeO_2_ NPs revealed high intensity peaks of Ce and O, as well as C, N, and Cl, which is from plant extract [46]. As shown in EDX results of CDV-CeO_2_ NPs, the strong band of P confirms the presence of surface loaded CDV on the structure of the green synthesized CeO_2_ NPs [48].

#### TEM images

**Figure 7:**
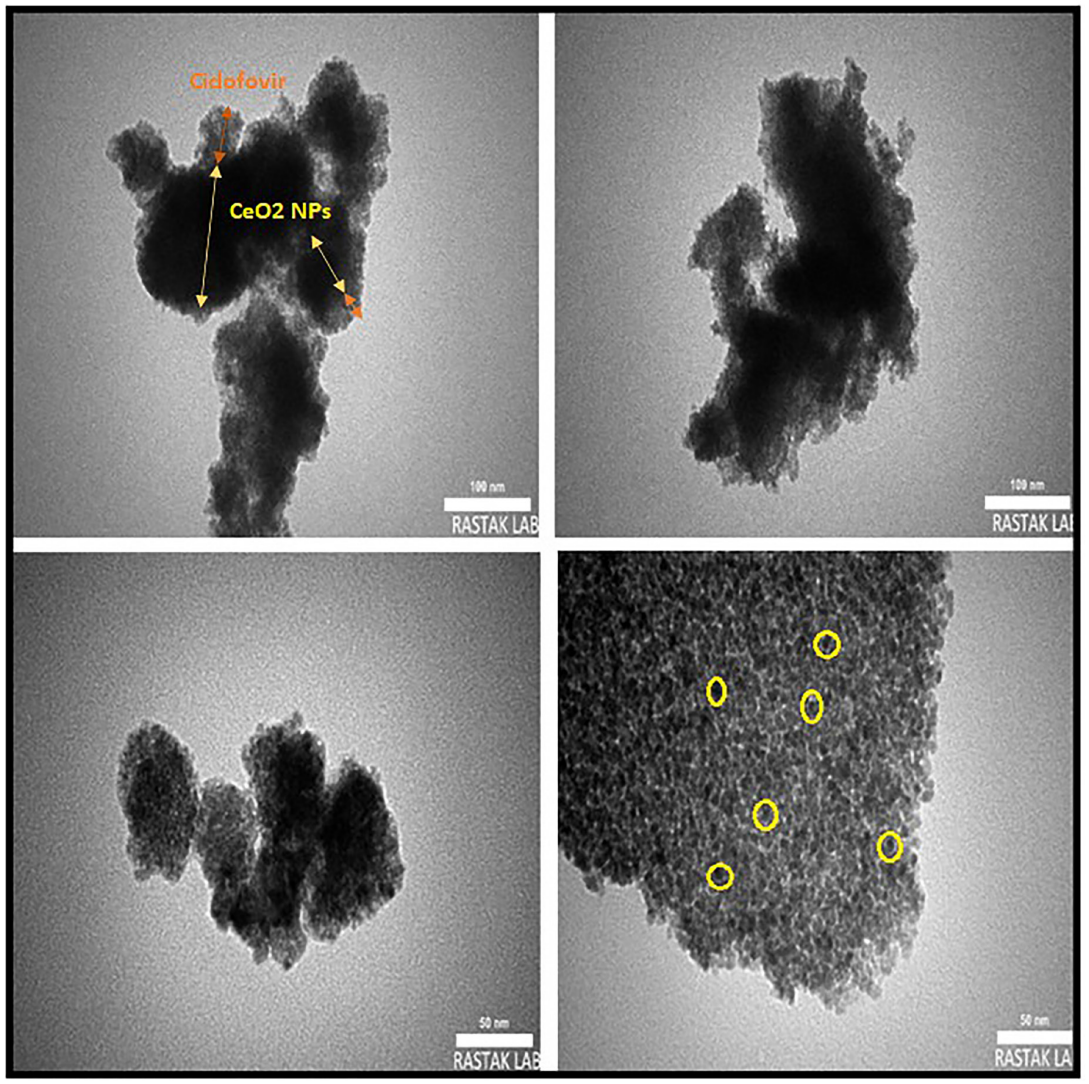
TEM images of CDV-CeO_2_ NPs with different magnification.

To detect the shape and size of the green-synthesized CeO_2_ nanoparticles, TEM technique was employed. The core-shell structure of the nanoparticles can be observed in Figure 7, where CeO_2_ NPs constitute the inner sphere, and the drug forms the outer shell. The TEM images revealed that the agglomerated NPs had a diameter size of 156.96 nm, while single NPs had a diameter of 14.90 nm. The CeO_2_ NPs agglomeration was observed to be high in aqueous media due to their high surface energy. In the image, the CeO_2_ NPs are depicted as the dark area within the core, whereas the drug is shown as a silver-gray outer shell.

#### Functional group analysis

**Figure 8:**
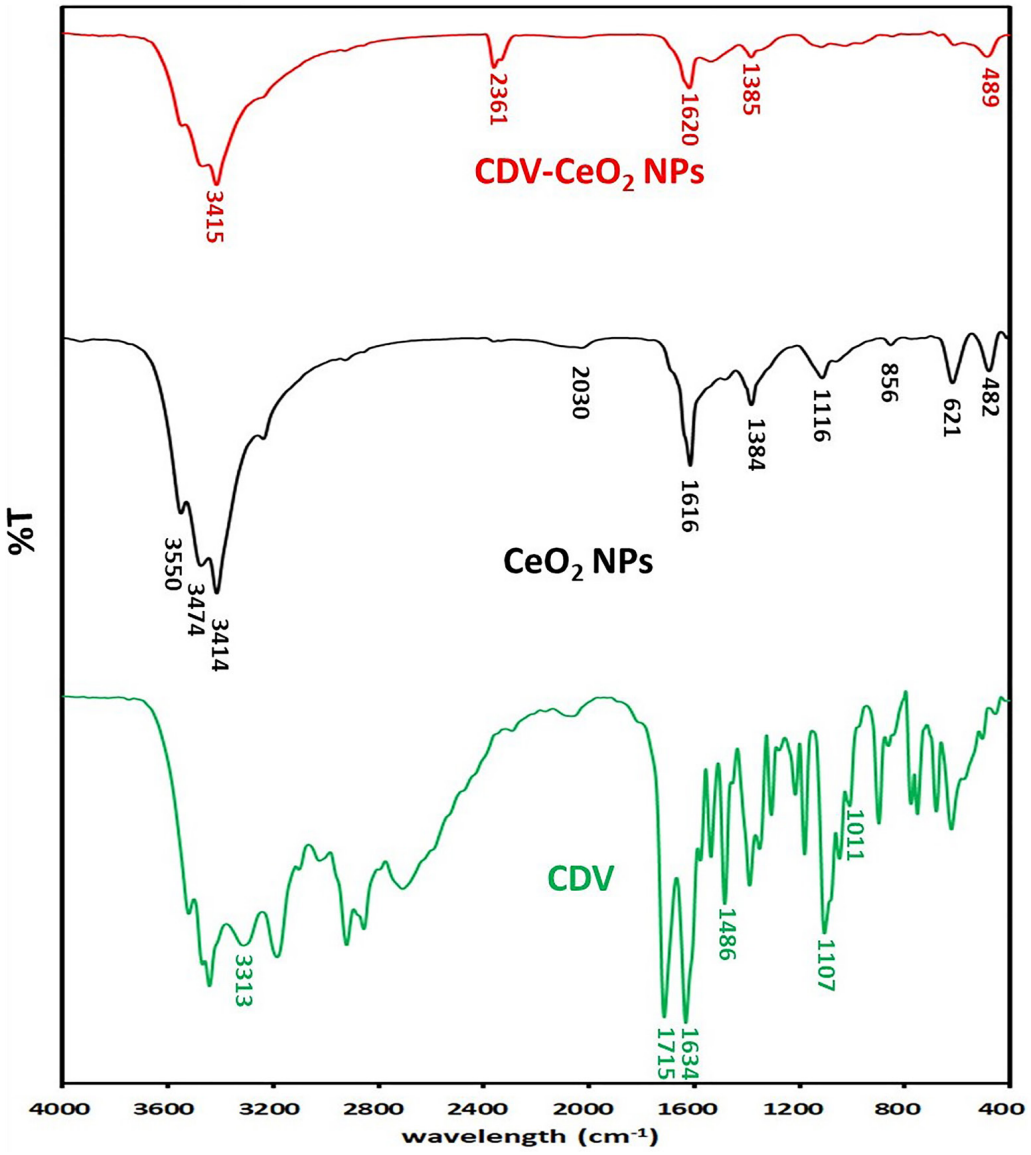
FTIR spectra of CDV, CeO_2_ NPs and CDV-CeO_2_ NPs.

Fourier transform infrared spectroscopy was used to identify the functional groups present in CDV, CeO_2_ NPs, and CDV-CeO_2_ NPs in the range of 500–4000 cm^−1^, and the results were tabulated in Figure 8. The broad absorption band observed at 3750–3000 cm^−1^ is due to the Ce-OH and O-H stretching from residual alcohols and water [49]. Our green synthesized CeO_2_ NPs showed absorption bands at 3550, 3474, and 3414 cm^−1^. Minor distortions in the 2030 cm^−1^ range indicate the presence of NH bonds in the Cydonia oblonga peel extract in the resulting CeO_2_ NPs [50]. The peaks observed at 1384 and 1616 cm^−1^ may be due to the C-H and C=O stretching vibrations of residual organic compounds, respectively [41]. The peak observed at 1116 cm^−1^ can be attributed to the overtone band of the trace of Ce-OH [51]. The bands at 482, 621, and 856 cm^−1^ can be correlated with the stretching frequency of Ce-O and the formation of CeO_2_ [52]. The FT-IR spectra of CDV show specific wavenumbers, namely 1715, 1634, 1486, 1011, and 1107 cm^−1^. The absorption peaks at 1107 and 1011 cm^−1^ correspond to the stretching vibration of P=O and P-O bonds, respectively. Additionally, the FT-IR spectra show the OH group of the cidofovir structure at 3313 cm^−1^ [53]. The FT-IR spectrum of CDV-CeO_2_ NPs demonstrates the characteristic peaks of CeO_2_ NPs and CDV, which is consistent with the formation of the CDV-CeO_2_ NPs structure [48].

### Cytotoxic assess

**Figure 9:**
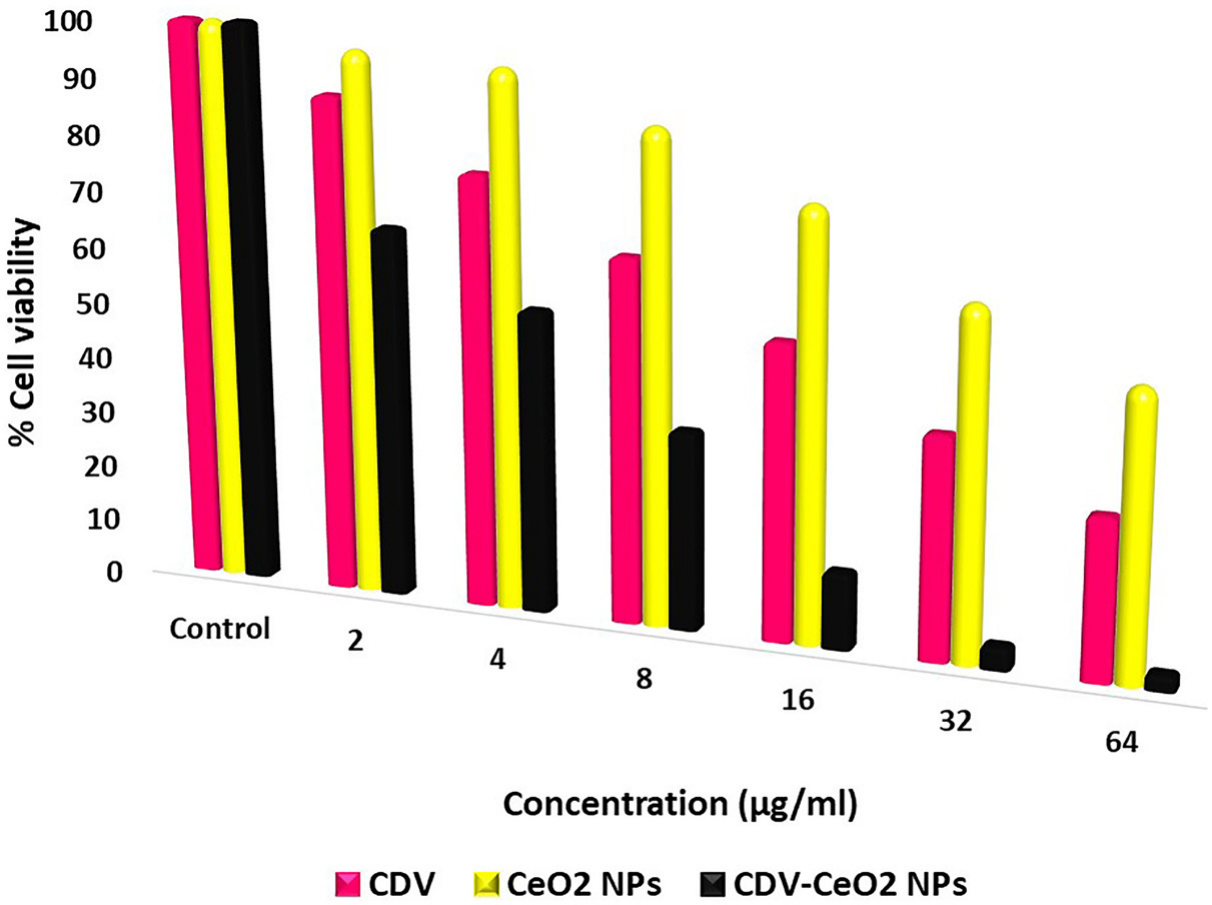
CDV, CeO_2_ NPs and CDV-CeO_2_ NPs on cell viability.

Figure 9 illustrates the inhibitory potential and sensitivity of CDV, CeO_2_ nanoparticles, and CDV-CeO_2_ nanoparticles against cancer cell growth at different concentrations. The anticancer activity increased with increasing treatment concentrations. At the highest concentration (64 μg/mL), the mortality rate of MCF-7 cancer cells was 71.65% for CDV, 49.85% for CeO_2_ nanoparticles, and 97.10% for CDV-CeO_2_ nanoparticles. The IC_50_ values were found to be 21.83 μg/mL, 40.11 μg/mL, and 4.46 μg/mL for CDV, CeO_2_ NPs, and CDV-CeO_2_ NPs, respectively. The results indicate that CDV-loaded CeO_2_ NPs exhibit more pronounced anticancer activity than CDV and CeO_2_ NPs alone. This suggests that loading CDV on CeO_2_ NPs leads to a significant enhancement of the anticancer and biological effects of CDV [54].

The results of this study are also comparable with the work conducted by Karampour et al. [55], in which magnetic γ-Fe_2_O_3_@SiO_2_ NPs loaded with CDV were synthesized and their cytotoxicity against MCF-7 cells was evaluated. At the highest tested concentration (160 μg/mL), the cell mortality rate for γ-Fe_2_O_3_@SiO_2_-CDV NPs was reported to be 75.84%, and the IC_50_ value was 74.26 μg/mL. In comparison, the CDV-CeO2 NPs synthesized in the present study exhibited significantly stronger anticancer effects at a lower concentration, with an IC_50_ value of only 4.46 ± 0.22 μg/mL approximately 17 times lower.

According to the obtained results, this compound can be considered a novel and effective candidate for the design of targeted drug delivery systems in breast cancer chemotherapy. However, to confirm the efficacy and safety of this nanocarrier for clinical applications, further studies including *in vivo* experiments, pharmacokinetic assessments, and detailed investigations of the underlying molecular mechanisms are necessary.

### Ct-DNA and RNA interaction

#### UV–vis spectroscopy

**Figure 10:**
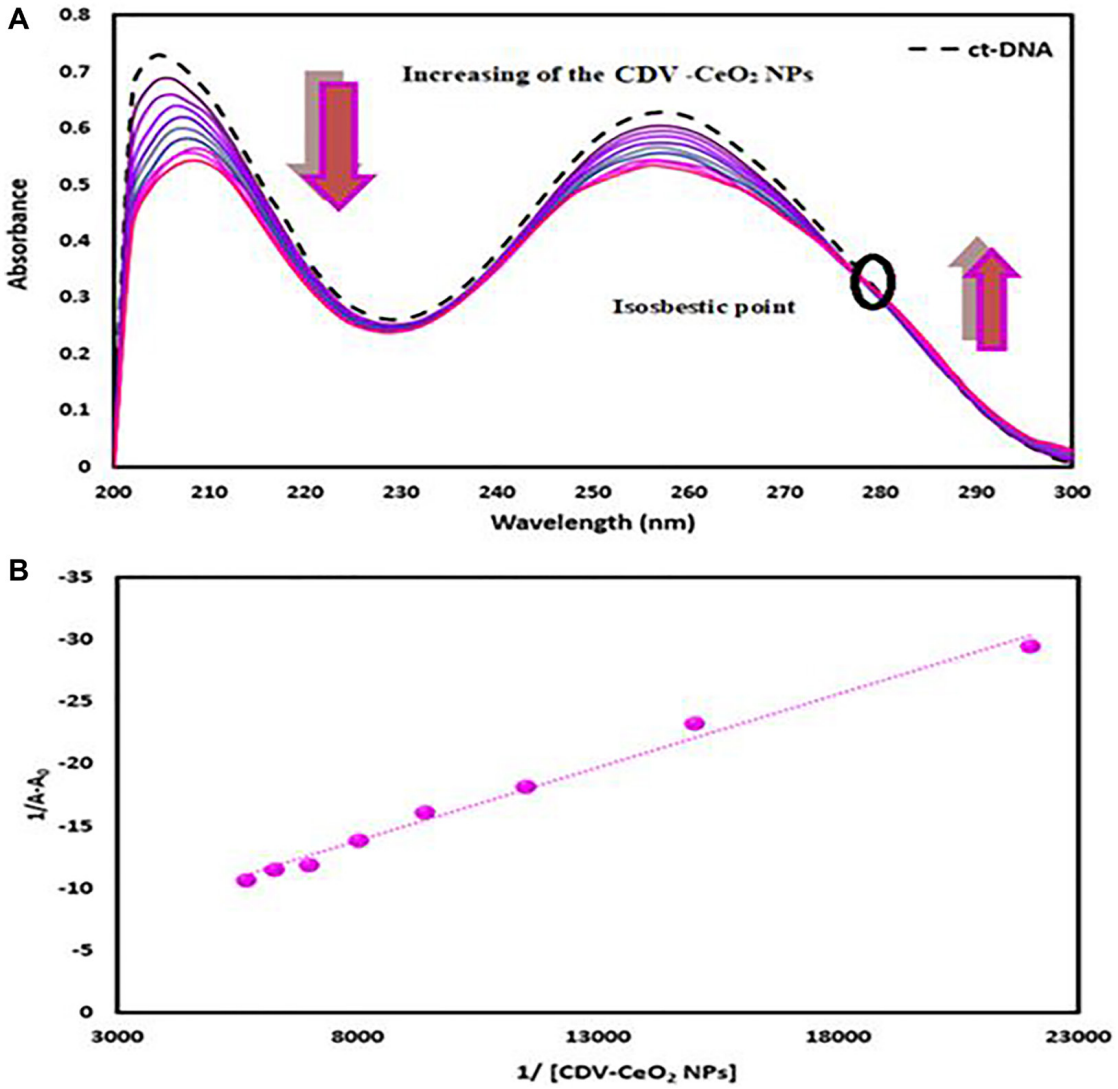
(**A**) Absorbance spectra of ct-DNA (4.00 × 10^−5^ M) with green synthesized CDV-CeO2 NPs (2.32 × 10^−5^ to 1.76 × 10^−4^ g/mL). (**B**) Wolfe-shimmer plot of ct-DNA- CDV-CeO2 NPs.

**Figure 11:**
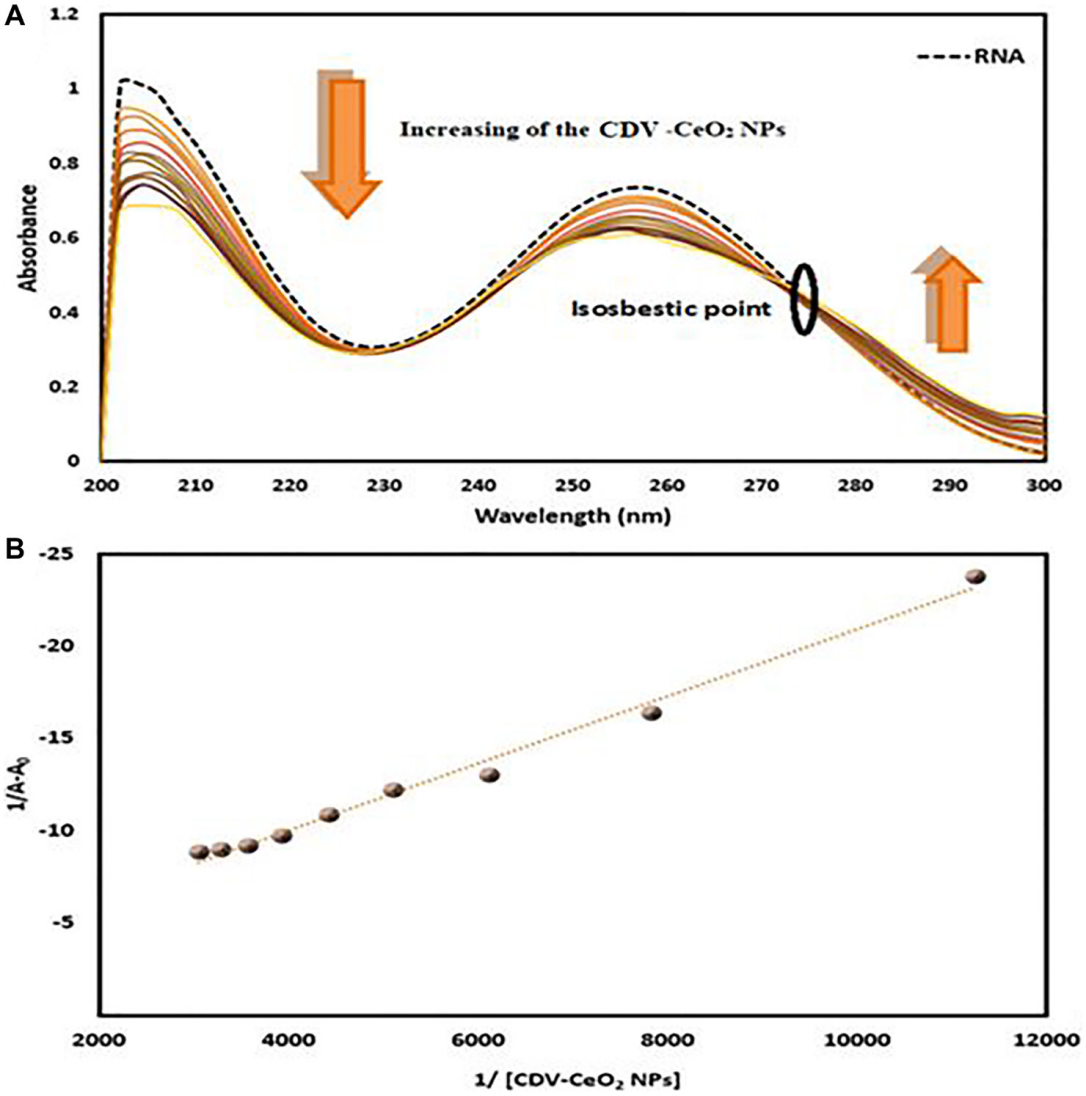
(**A**) Absorbance spectra of RNA (4.00 × 10^−5^ M) with green synthesized CDV-CeO_2_ NPs (2.32 × 10^−5^ to 1.76 × 10^−4^ g/mL). (**B**) Wolfe-shimmer plot of RNA- CDV-CeO_2_ NPs.

To assess the formation of CDV-CeO_2_ NPs-DNA and CDV-CeO_2_ NPs-RNA complexes, the ultraviolet-visible absorption spectra of DNA/RNA with and without CDV-CeO_2_ NPs were examined. Figures 10A and 11A indicate that the DNA/RNA absorption spectra exhibited hypochromism, which resulted from the formation of a ground-state complex with CDV-CeO_2_ NPs. Furthermore, the isosbestic point indicated the formation of a new CDV-CeO_2_ NPs-nucleic acid complex, demonstrating that the binding of CDV-CeO_2_ NPs to DNA and RNA indeed occurred [56].

The Wolfe–Shimmer equation was used to calculate the association constant of the nucleic acids and CDV-CeO_2_ NPs interaction:


$$1/(\text{A}-{\text{A}}_{0})=1/(\text{A}-{\text{A}}_{0})+1/{\text{K}}_{\text{b}}({\text{A}}_{\infty }-{\text{A}}_{0}).1/(\text{CDV}-{\text{CeO}}_{2}\;\text{NPs})\;(2)$$

Where, A_0_ is the free nucleic acids ‘absorbance at 260 nm, and A is the absorbance of biomolecule with CDV-CeO_2_ NPs. To calculate the binding constant (K_b_) of biomolecule-CDV-CeO_2_ NPs system the plot of 1/(A-A_0_) versus 1/(CDV-CeO_2_ NPs) was employed (see Figures 10B and 11B). The obtained K_b_ value was 3.63 × 10^3^ and 1.26 × 10^3^ M (M = g/mL) for DNA and RNA, respectively. The spectrofluorimetry technique can give a mechanistic detail of the exact nucleic acids - CDV-CeO_2_ NPs system’ binding mode. Thus, it was used to explore the mode of binding in this study.

#### Fluorescence studies

**Figure 12:**
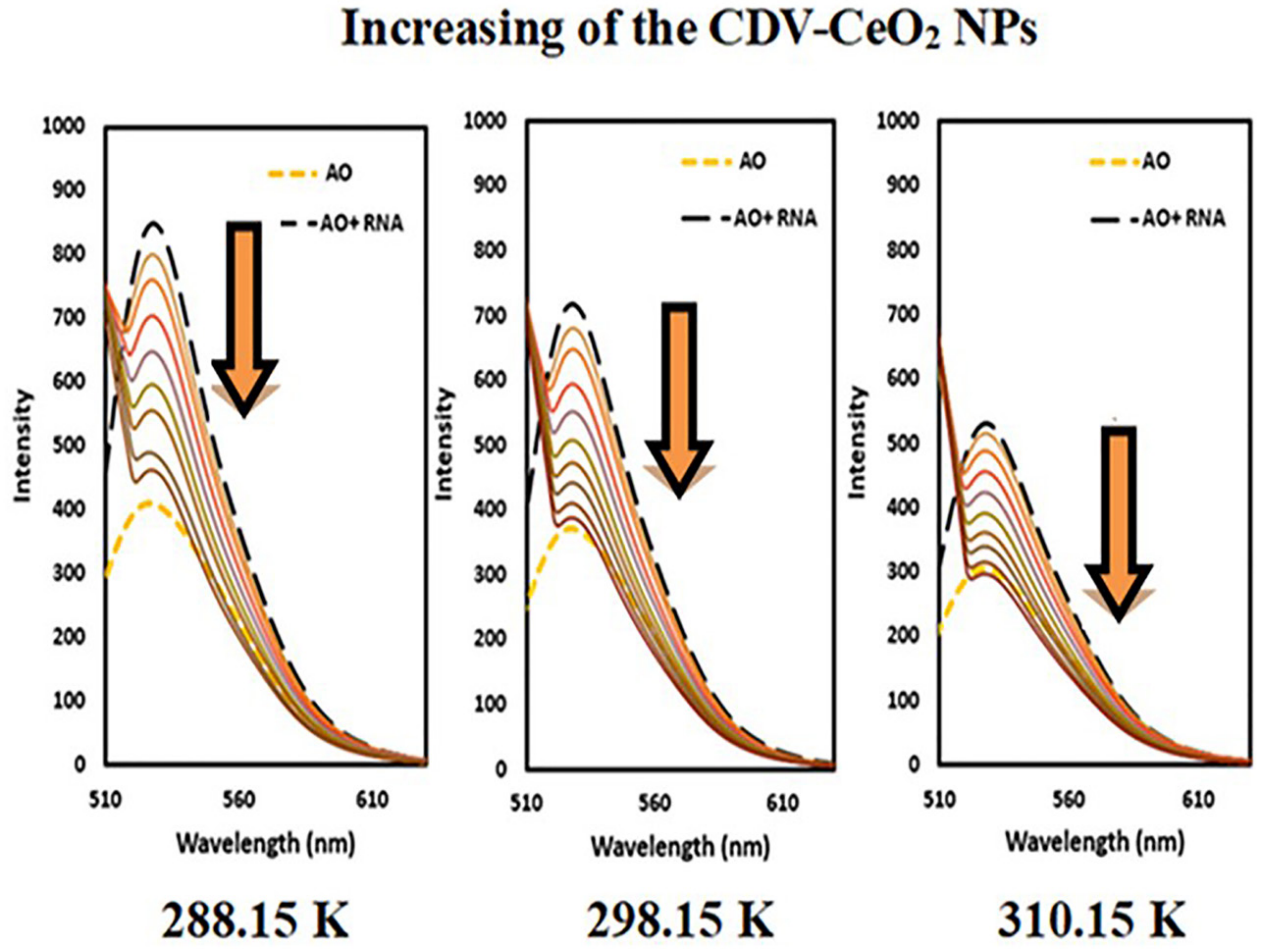
Fluorescence spectra of AO + RNA in the presence of CDV-CeO_2_ NPs (2.44 × 10^−5^ to 2.59 × 10^−4^ g/mL), RNA = (1.76 × 10^−4^ M) C_AO_ = (5.00 × 10^−6^ M).

**Figure 13:**
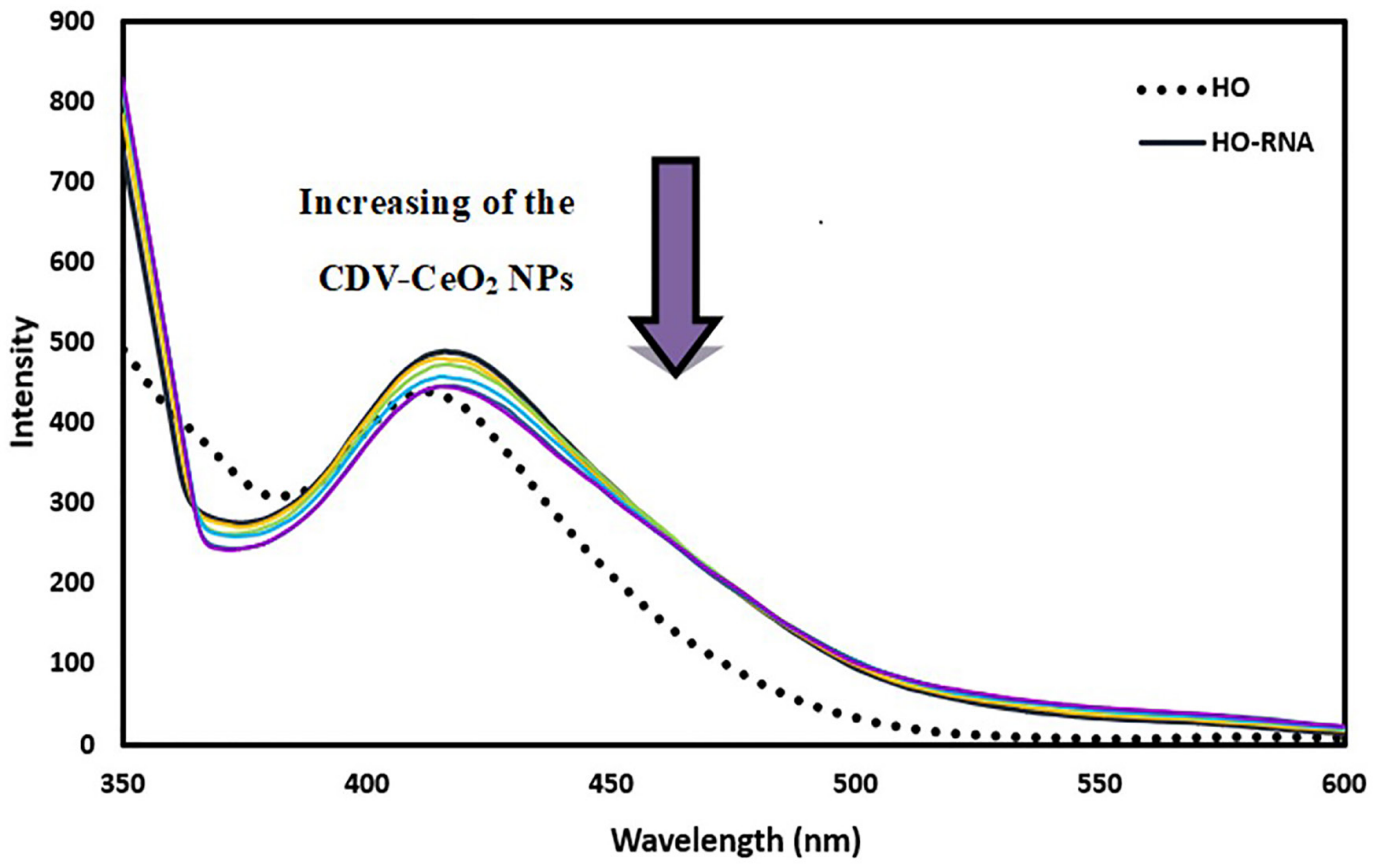
Fluorescence spectra of Hoechst + RNA in the presence of CDV-CeO_2_ NPs (2.44 × 10^−5^ to 2.59 × 10^−4^ g/mL), RNA = (1.76 × 10^−4^ M) C_Hoechst_ = (5.00 × 10^−6^ M).

Fluorescence method was employed to further assess the interactions of nucleic acids with CDV-CeO_2_ NPs. DNA and RNA alone exhibit a weak fluorescence signal because of their fluoresce quenching by solvent molecules. Also, in aqueous solution, AO and HO exhibit low fluorescence in their free state but show an increase in emission intensity upon interaction with DNA/RNA. The emission intensity of RNA-AO and RNA-HO system was recorded on subsequent addition in enhancing amounts of CDV-CeO_2_ NPs. The RNA-AO system’ emission intensity decreased upon elevating the CDV-CeO_2_ NPs different concentrations (see Figure 12). This result indicated that CDV-CeO_2_ NPs block the site for bonding of AO, that is, they have the same binding region on RNA. As can be seen, RNA represents a weak interaction with HO and the there is a negligible enhancement in the fluorescence intensity of HO in the presence of RNA (see Figure 13). This is believed to be due to the shallow minor grooves of RNA. Additionally, the addition of CDV-CeO_2_ NPs to the HO-RNA solution did not result in any significant changes in fluorescence intensity. Therefore, we conducted fluorescence measurements at three different temperatures (288.15, 298.15, 310.15 K) by using AO to determine the binding constant.

**Figure 14:**
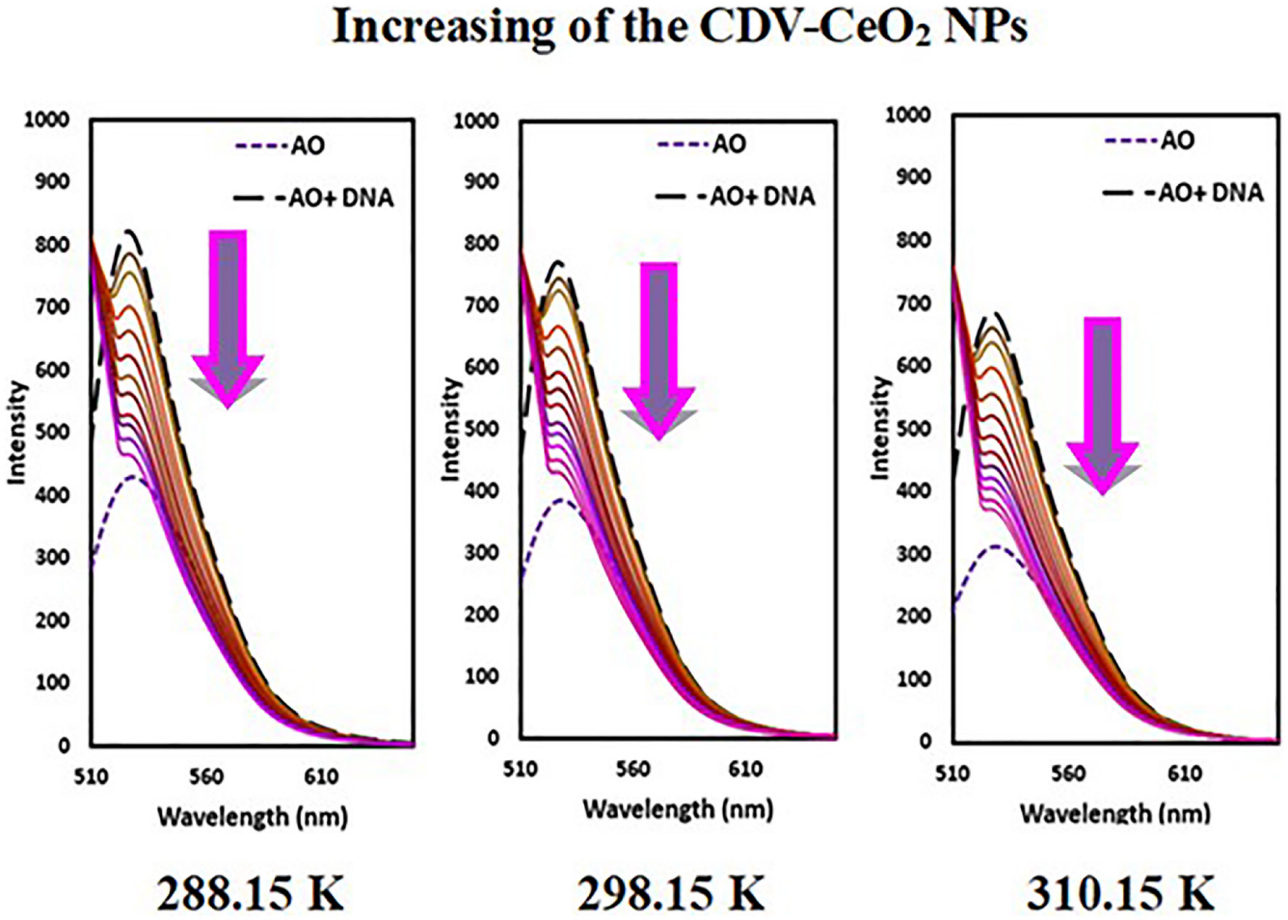
Fluorescence spectra of AO + DNA in the presence of CDV-CeO_2_ NPs (2.44 × 10^−5^ to 2.59 × 10^−4^ g/mL), DNA = (1.76 × 10^−4^ M) C_AO_ = (5.00 × 10^−6^ M).

**Figure 15:**
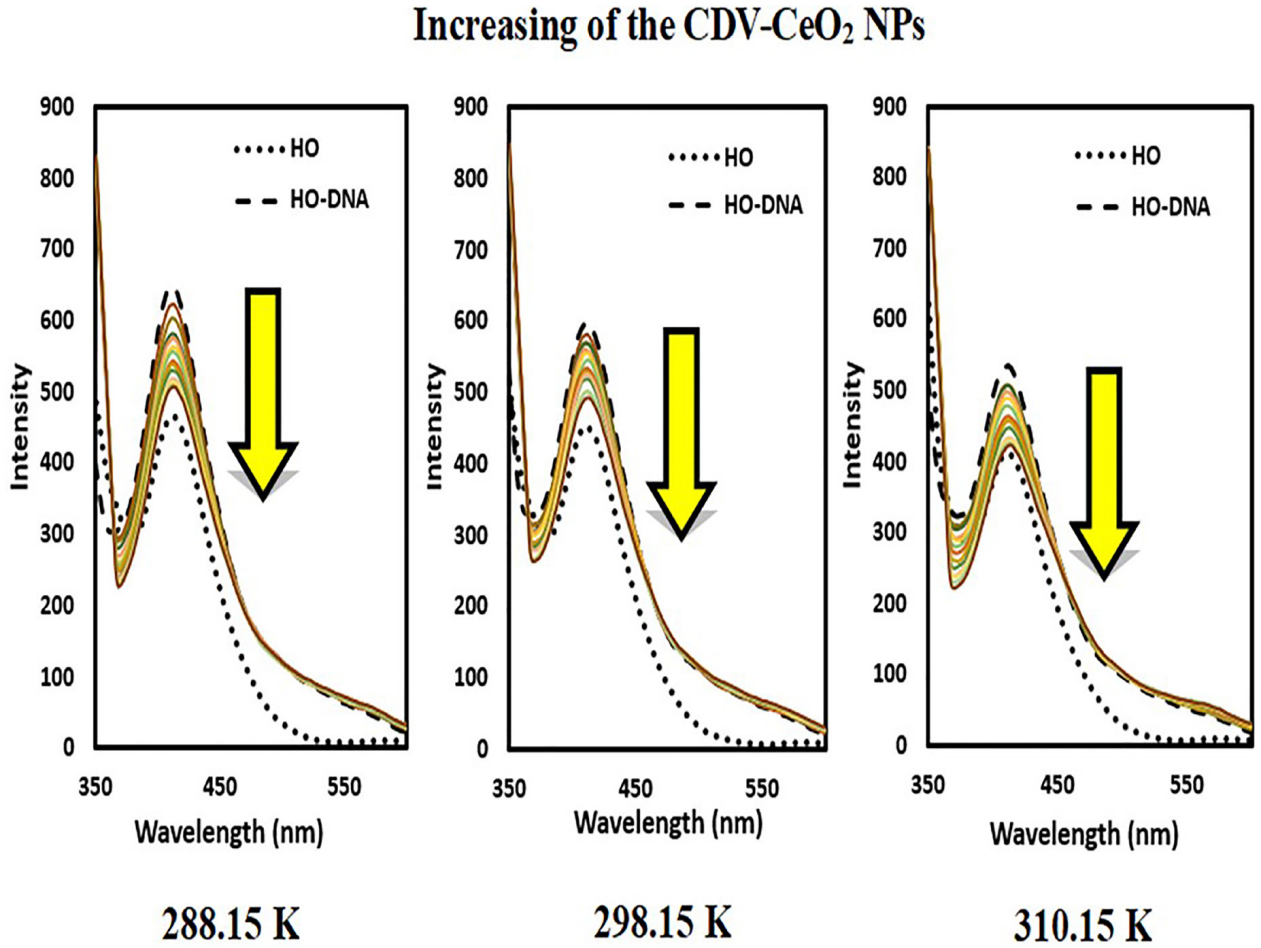
Fluorescence spectra of Hoechst + ct-DNA in the presence of CDV-CeO_2_ NPs (2.44 × 10^−5^ to 2.59 × 10^−4^ g/mL), Ct-DNA = (1.76 × 10^−4^ M) C_Hoechst_ = (5.00 × 10^−6^ M).

The emission intensity of DNA-AO and DNA-HO system was recorded on subsequent addition in enhancing amounts of CDV-CeO_2_ NPs. As Figures 14 and 15 show, the emission intensity of the systems (DNA-AO and DNA-HO) is strongly affected by different concentrations of CDV-CeO_2_ NPs. The DNA-AO system’ emission intensity decreased upon elevating the CDV-CeO_2_ NPs ‘ different concentrations. This result indicated that CDV-CeO_2_ NPs intercalated to DNA helix, thereby block the site for bonding of AO. Besides, with addition, of various amounts of CDV-CeO_2_ NPs the DNA-HO ‘ emission intensity decreased, showing that CDV-CeO_2_ NPs remove HO from the DNA grooves. So, analysis of the data show that CDV-CeO_2_ NPs interact with DNA both through the groove and intercalate (partial intercalation) [57]. Therefore, we conducted fluorescence measurements in the presence of both AO and HO at three distinct temperatures (288.15, 298.15, and 310.15 K) to calculate the binding constant.

To determine whether the quenching mechanism of the CDV-CeO_2_ NPs/labeled nucleic acids system is static or dynamic in nature, we utilized the following Stern-Volmer equation (Eq. 3) [58]:


$${\text{F}}_{0}/\text{F}=1+{\text{K}}_{\text{SV}}\;(\text{Q})=1+\tau_{\text{o}}\;\text{kq}\;(3)$$

**Figure 16:**
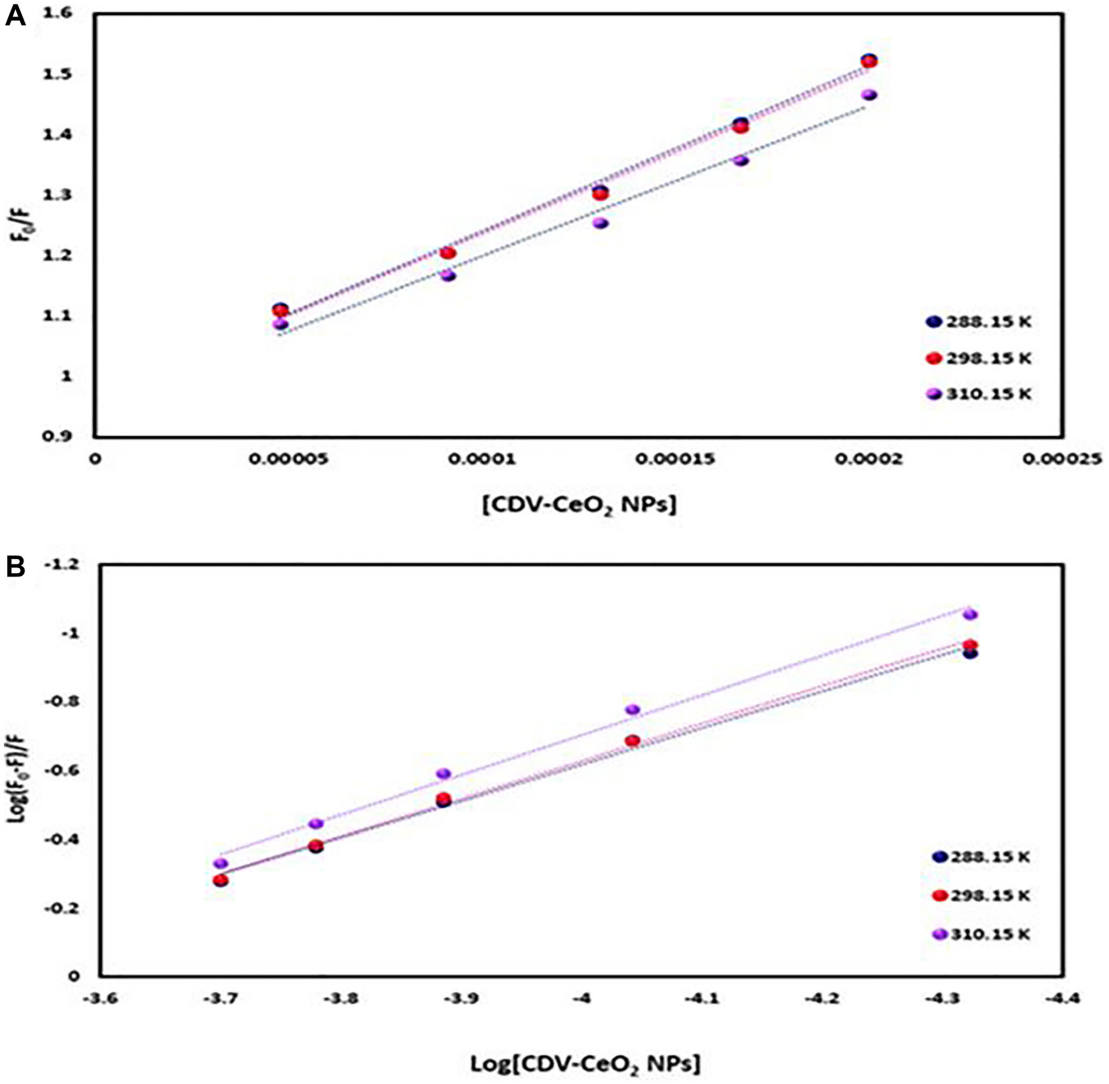
(**A**) Plot of F_0_/F versus (CDV-CeO_2_ NPs) for RNA–CDV-CeO_2_ NPs system (RNA was labeled with AO). (**B**) Plot of log (F_0_-F)/F versus log (CDV-CeO_2_ NPs) for RNA–CDV-CeO_2_ NPs system (RNA was labeled with AO).

**Figure 17:**
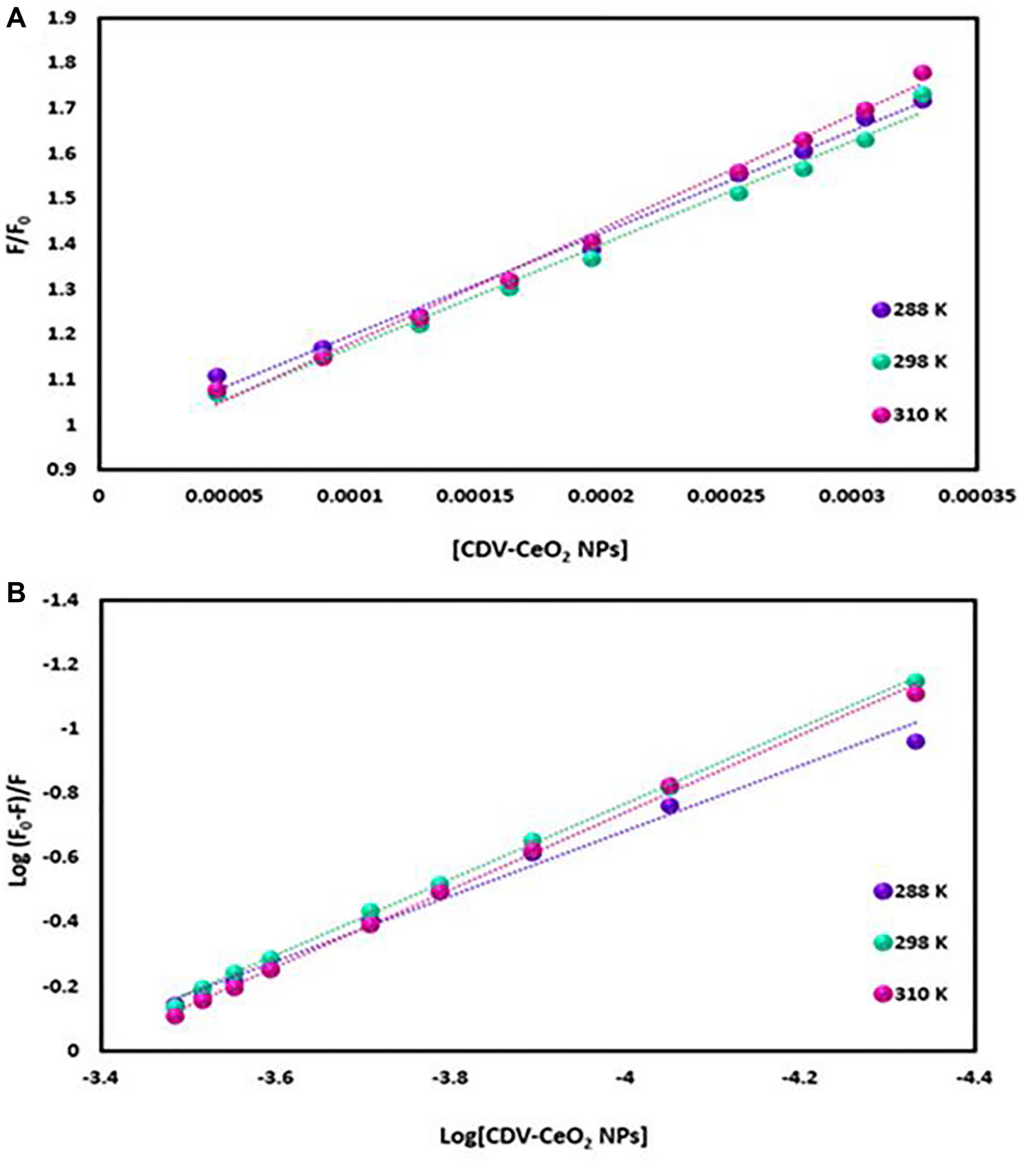
(**A**) Plot of F_0_/F versus (CDV-CeO_2_ NPs) for ct-DNA–CDV-CeO_2_ NPs system (ct-DNA was labeled with AO). (**B**) Plot of log (F_0_-F)/F versus log (CDV-CeO_2_ NPs) for ct-DNA–CDV-CeO_2_ NPs system (ct-DNA was labeled with AO).

**Figure 18:**
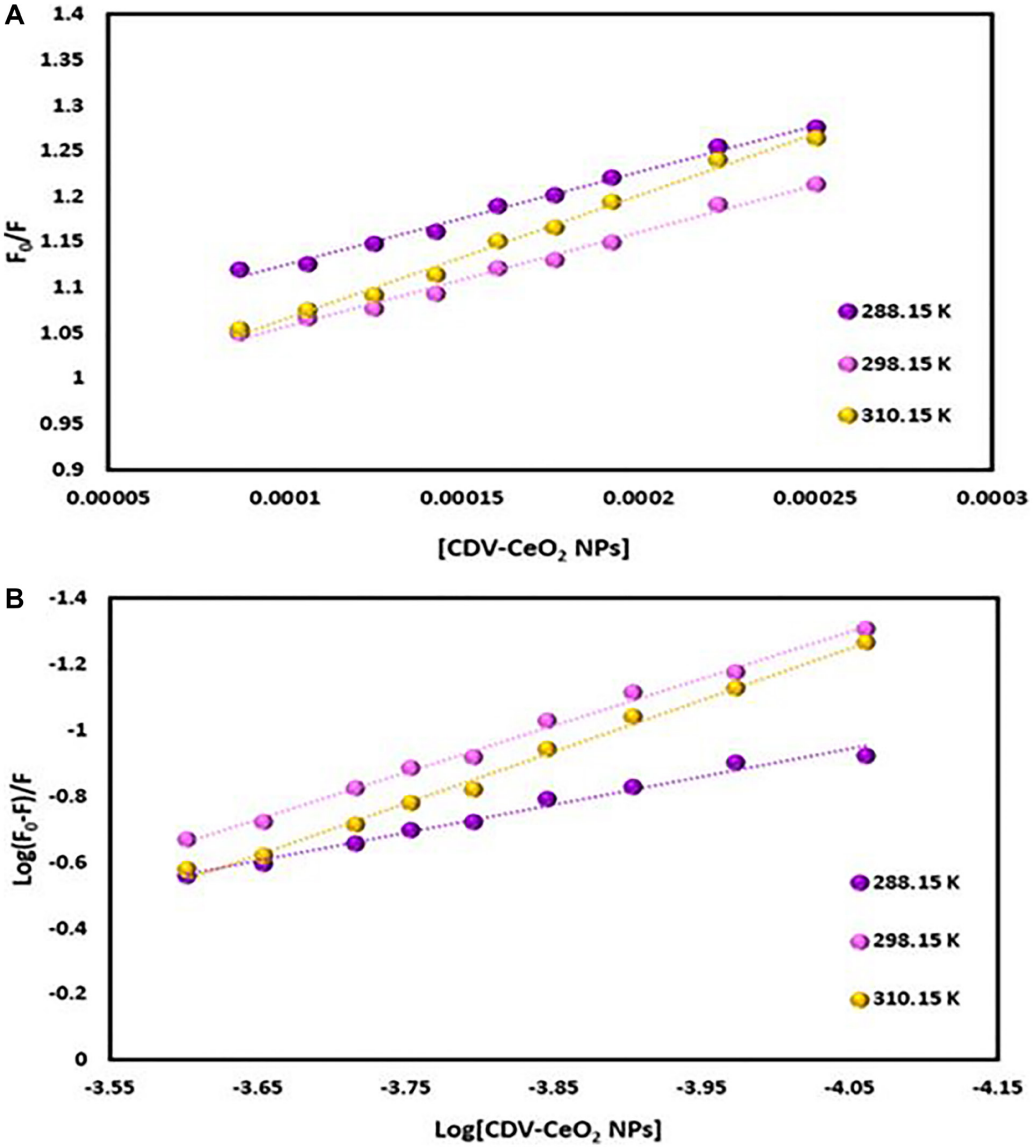
(**A**) Plot of F_0_/F versus (CDV-CeO_2_ NPs) for ct-DNA–CDV-CeO_2_ NPs system (ct-DNA was labeled with HO). (**B**) Plot of log (F_0_-F)/F versus log (CDV-CeO_2_ NPs) for DNA–CDV-CeO_2_ NPs system (ct-DNA was labeled with HO).

The emission intensity of labeled nucleic acids in the absence and presence of CDV-CeO_2_ NPs is represented by F_0_ and F, respectively. τ_o_ is the labeled nucleic acids’ lifetime in the absence of CDV-CeO_2_ NPs, (Q) is the concentration of CDV-CeO_2_ NPs, Ksv is the Stern-Volmer quenching constant, and kq is the apparent bimolecular quenching rate constant, which is calculated based on Ksv = kq τ_o_ [59]. The Stern-Volmer quenching constant was obtained from the slope of the plot of F_0_/F vs. (CDV-CeO_2_ NPs) (see Figures 16A, 17A and 18A). Since the interaction of RNA with the CDV-CeO_2_ NPs in the presence of HO was not satisfactory, we calculated the binding constant for the RNA + AO system with increased CDV-CeO_2_ NPs. For RNA the values of K_sv_ showed decreased with the temperature rise (see Table 1), revealed that static quenching was the primary process involved in the complex formation [60]. For DNA-AO and DNA-HO systems, the K_sv_ values increased on rising the temperature (see Tables 2 and 3), which implied that dynamic quenching is the main process involved in the complex formation. Dynamic quenching only impacts the excited state of the quenching molecule and does not alter the absorption spectrum of DNA. On the other hand, in static quenching, a complex of DNA and ligand is formed, resulting in alterations in the UV-visible spectra of DNA. The results of the UV-Vis experiment (see Figure 10A) further confirmed that static quenching is the primary quenching mechanism for the DNA-CDV-CeO2 NPs complex [61].

**Table 1:** Binding and thermodynamic parameters of the RNA–CDV-CeO_2_ NPs system (RNA was labeled with AO)

T(K)	K_sv_ (M^−1^)^*^	K_q_ (M^−1^S^−1^)^*^	K_b_ (M^−1^)^*^	*n*	ΔG^0^ (kJmol^−1^)	ΔH^0^ (kJmol^−1^)	ΔS^0^ (Jmol^−1^K^−1^)
288.15	**2.72 × 10^3^**	**2.72 × 10^11^**	**4.37 × 10^3^**	**1.06**	**−19.99**	**21.62**	**144.42**
298.15	**2.69 × 10^3^**	**2.69 × 10^11^**	**5.31 × 10^3^**	**1.09**	**−21.43**		
310.15	**2.48 × 10^3^**	**2.48 × 10^11^**	**8.25 × 10^3^**	**1.15**	**−23.17**		

^*^M = g/mL.

**Table 2:** Binding and thermodynamic parameters of the ct-DNA–CDV-CeO_2_ NPs system (ct-DNA was labeled with AO)

T(K)	K_sv_ (M^−1^)^*^	K_q_ (M^−1^S^−1^)^*^	K_b_ (M^−1^)^*^	*n*	ΔG^0^(kJmol^−1^)	ΔH^0^(kJmol^−1^)	ΔS^0^(Jmol^−1^K^−1^)
288.15	**2.26 × 10^3^**	**2.23 × 10^11^**	**2.26 × 10^3^**	**1.00**	**−18.99**	**53.41**	**251.26**
298.15	**2.28 × 10^3^**	**2.28 × 10^11^**	**8.65 × 10^3^**	**1.18**	**−21.50**		
310.15	**2.52 × 10^3^**	**2.52 × 10^11^**	**1.12 × 10^4^**	**1.20**	**−24.52**		

^*^M = g/mL.

**Table 3:** Binding and thermodynamic parameters of the ct-DNA–CDV-CeO_2_ NPs system (ct-DNA was labeled with HO)

T(K)	K_sv_ (M^−1^)^*^	K_q_ (M^−1^S^−1^)^*^	K_b_ (M^−1^)^*^	*n*	ΔG^0^(kJmol^−1^)	ΔH^0^(kJmol^−1^)	ΔS^0^(Jmol^−1^K^−1^)
288.15	**1.02 × 10^3^**	**1.02 × 10^11^**	**3.26 × 10^2^**	**0.85**	**−36.02**	**197.01**	**736.74**
298.15	**1.04 × 10^3^**	**1.04 × 10^11^**	**2.85 × 10^4^**	**1.42**	**−34.95**		
310.15	**1.36 × 10^3^**	**1.36 × 10^11^**	**1.19 × 10^5^**	**1.56**	**−33.66**		

^*^M = g/mL Figure legends.

Determination of the binding constant (K_b_) and number of binding sites (n) can be done using the following equation:


$$\text{Log}(({\text{F}}_{0}-\text{F})/\text{F})=\log\;{\text{K}}_{\text{b}}+n\;\log(\text{CDV}-{\text{CeO}}_{2}\;\text{NPs})\;(4)$$

K_b_ and n values are from the intercept and slope of the plot of log (F_0_-F)/F versus log (CDV-CeO_2_ NPs) (see Figures 16B, 17B and 18B). The values of K_b_ and n are given in (see Tables 1, 2 and 3). Since the value of n for all of the RNA and DNA systems was close to 1, it indicates that there is a binding site for the CDV-CeO_2_ NPs on the RNA and DNA. For all of the RNA and DNA systems the K_b_’ values increase by temperature enhancing reveals that the CDV-CeO_2_ NPs–nucleic acids complexes increase their stability on elevating temperature.

### Thermodynamic parameters and interaction forces

To determine the thermodynamic binding parameters (ΔH^0^ and ΔS^0^), the researchers utilized the van’t Hoff equation (Eq. 5) as follows:


$$\text{Ln}\;{\text{K}}_{\text{b}}=-\Delta {\text{H}}^{0}/\text{RT}+\Delta {\text{S}}^{0}/\text{R}\;(5)$$

The calculation of ΔH^0^ and ΔS^0^ can be achieved by using the slope and intercept of a linear van’t Hoff plot. Gibbs free energy change (ΔG^0^) is determined as:


$$\Delta {\text{G}}^{0}=\Delta {\text{H}}^{0}-\text{T}\Delta {\text{S}}^{0}\;(6)$$

Spontaneous binding is authenticated by the value of ΔG^0^ that is negative. The enthalpy and entropy positive values (see Table 1, 2 and 3) suggest that hydrophobic interactions are taken part in the nucleic acids - CDV-CeO_2_ NPs complex formation [62].

## MATERIALS AND METHODS

### Materials

**Figure 19:**
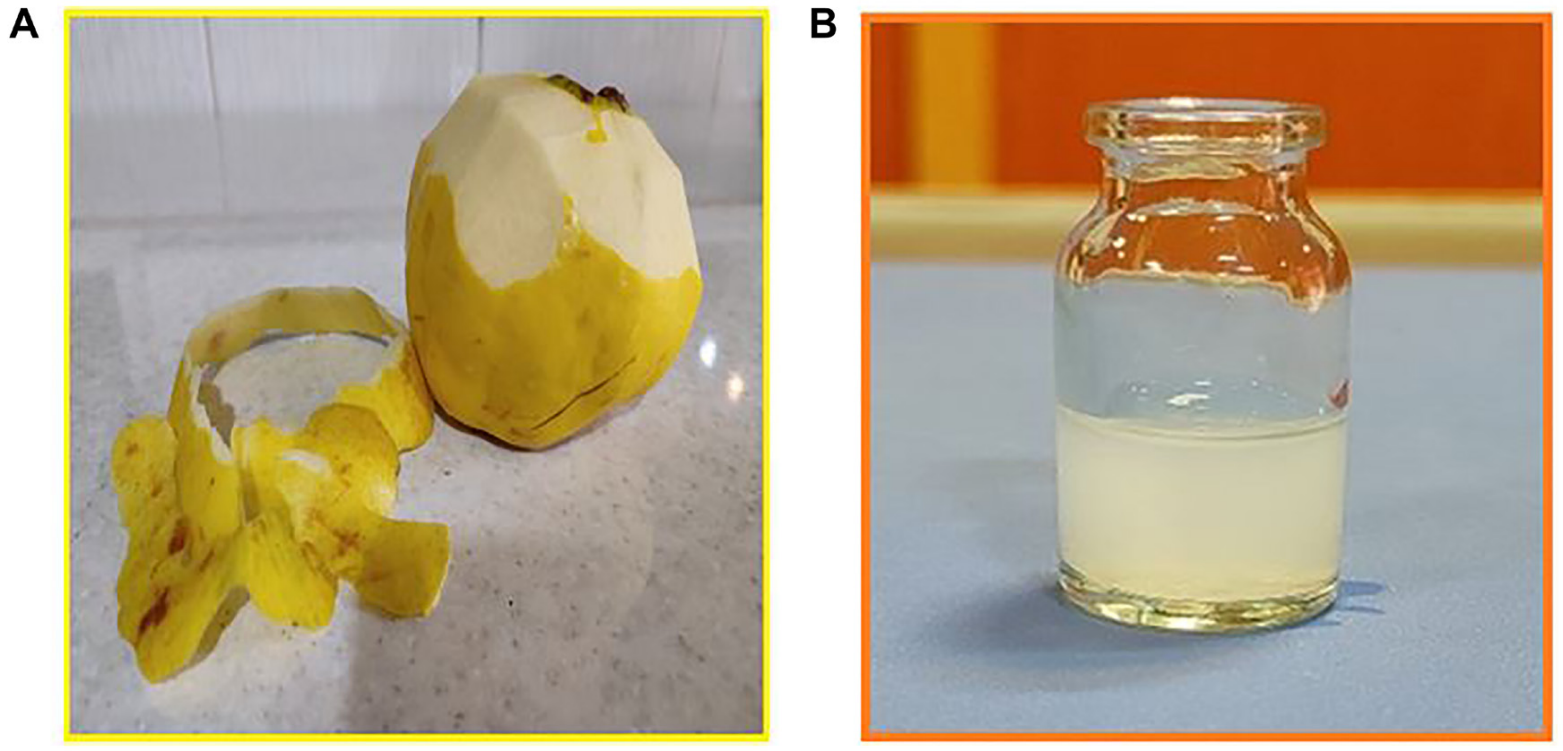
(**A**) The quince fruit and the peel of the quince fruit (**B**) digital photograph of CeO_2_ NPs were dispersed in water.

To synthesize CeO_2_ nanoparticles using a green approach, Ce (NO_3_)_3_. 6H_2_O salt was used. CDV was used for drug loading, while the MTT assay employed 3-(4, 5-dimethylthiazol-2-yl)-2, 5-diphenyl tetrazolium bromide (MTT), Fetal bovine serum, DMEM (Dulbecco Modified Eagles Medium), streptomycin, and penicillin. To investigate interactions, Tris- (hydroxymethyl)-amino-methane–hydrogen chloride, Hoechst 33258 (HO), Acridine orange (AO), Baker’s yeast RNA sodium salt, and calf thymus DNA were provided from reputable companies. To create a Tris-HCl buffer solution with a pH of 7.4, Tris-(hydroxymethyl)aminomethane was dissolved in distilled water. For interaction experiments, the stock solution of RNA and DNA was prepared by dissolving a predetermined amount of pure RNA and DNA powder in Tris–HCl buffer. To assess the purity of the RNA and DNA solution, we measured the UV absorbance values at 260 and 280 nm (A_260_/A_280_), which yielded a value of 1.88. The HO and AO solution (1.00 × 10^−3^ M) was provided in distilled water. The CeO_2_ nanoparticles solution with a concentration of 1 mg/ml was provided by sonicating the NPs powder in distilled water for 10 minutes (refer to Figure 19B).

### Preparation of the quince fruit (Cydonia oblonga) peel extract

Fruits of quince (*Cydonia oblonga*) were purchased and the peels of the fruit were taken (see Figure 19A). After that, the peels were cleaned by distilled water, then they were air-dried under shade for 5–7 days and grinded to obtain fine powdered form. The resulting powder was immersed in 100 mL of deionized water after grinding, and the mixture was boiled at 80°C for 30 minutes to obtain a concentrated yellowish-brown extract. After cooling to room temperature, the extract was filtered employing Whatman filter paper (41 pore size) and subsequently stored.

### Preparation and characterization of NPs

**Figure 20:**
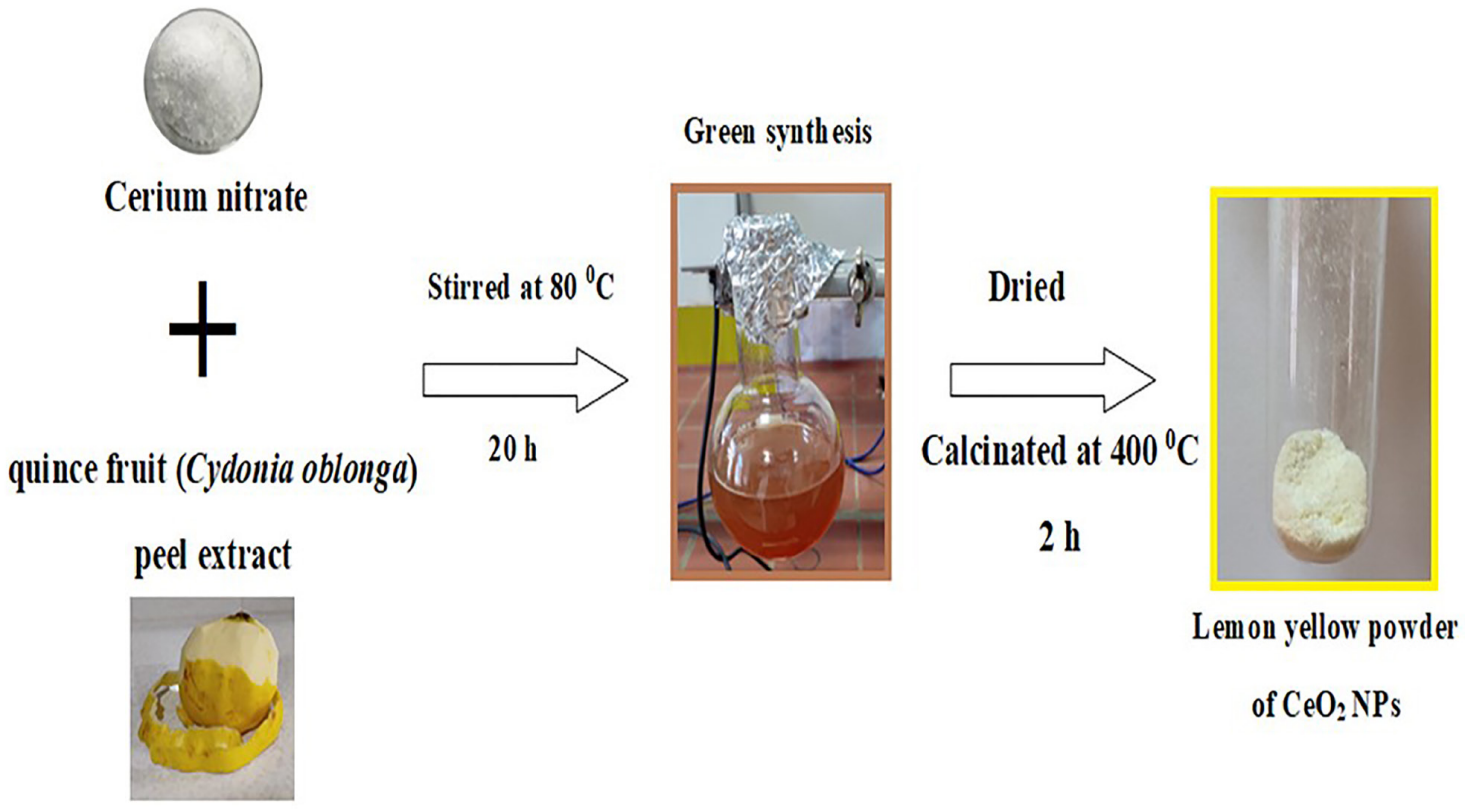
A schematic diagram for the generation of CeO_2_ NPs using *Cydonia oblonga* peel extract.

To generate CeO_2_ NPs using Cydonia oblonga peel extract, 3.72 g of Ce (NO_3_)_3_.6H_2_O salt was added to the extract and gently stirred at 80°C for 20 hours. The process continued until the formation of a white precipitate. The precipitate gradually turned yellowish-brown on continuous stirring. The received precipitate was then calcined at 400°C for 2 hours, resulting in a yellow powder of CeO_2_ NPs (loss of H_2_O to form Ce(IV) oxide nanoparticles). Figure 20 shows a schematic diagram of the generation of CeO_2_ NPs employing this method. The generation of NPs was confirmed through FT-IR, zeta potential, TEM, SEM-EDX, DLS, and ultraviolet-visible analysis.

### Loading of CDV on the structure of the CeO_2_ NPs

The CeO_2_ nanoparticles solution (1 mg/ml) was initially sonicated in double-distilled water for 20 minutes at room temperature. Subsequently, the same solution was mixed with CDV solution (1 mg/mL) in equal volumes by stirring for 24 hours at room temperature. The resulting mixture was then centrifuged at 7000 rpm for 20 minutes to obtain CDV-CeO_2_ nanoparticles. To assess the drug loading efficiency, the supernatant obtained from the centrifugation was subjected to spectrophotometry to determine the absorbance at 249 nm for unloaded drug. The calculation of the drug loading efficiency involved subtracting the quantity of unbound drug in the supernatant from the total amount of added drug. The standard curve equation was used to determine the drug loading efficiency, which was estimated using the following equation:


$$\text{Loading efficiency}=\frac{\text{Total Drug}-\text{Unloaded Drug}(\text{in supernatant})}{\text{Total Drug}}\times 100\;(7)$$

### Cell culture conditions

To investigate the potential inhibitory effects of CDV, CeO_2_ NPs, and CDV-CeO_2_ NPs on cancer cell lines, MCF-7 cells were used. The cells were cultured in DMEM containing 200 mM L-glutamine, 10% fetal bovine serum (FBS), 100 μg/mL penicillin, and 10 mg/mL streptomycin. The cells were maintained under a 5% CO_2_ atmosphere at 37°C.

#### *In vitro* cytotoxicity (MTT assay)

The standard MTT assay was done to evaluate the anticancer potential of CDV, CeO_2_ NPs, and CDV-CeO_2_ NPs on MCF-7 cancer cells, following the methodology described in our previous studies [43, 63]. To begin, a predetermined number of cells (1 × 10^4^ cells) were seeded in a 96-well plate and incubated for 24 hours at 37°C. The cells were then treated with selected concentrations (2, 4, 8, 16, 32, and 64 μg/mL) of CDV, CeO_2_ NPs, and CDV-CeO_2_ NPs for 24 hours. Next, MTT was added to each well and incubated for 4 hours. After incubation, 200 μL of DMSO solution was added to each well and the plate was kept for 10 minutes. The plate absorbance was noted at 550–570 nm using an Elisa microplate reader.

### Nucleic acids (DNA and RNA) binding affinity

#### UV-Vis spectroscopy

The UV-visible spectra of RNA and DNA were noted alone and in the presence of CDV-CeO_2_ NPs. The measurement was taken across the wavelength range of 200 to 400 nm. A constant concentration of RNA and DNA (40 μM) was titrated with varying concentrations (2.32 × 10^−5^ to 1.76 × 10^−4^ g/mL) of CDV-CeO_2_ NPs, and baseline correction was performed using Tris-HCl buffer.

#### Fluorescence studies

##### Evaluating association mode

The groove binders (HO) and the intercalator fluorescence probe (AO) were utilized to elucidate the DNA-CDV-CeO_2_ NPs and RNA-CDV-CeO_2_ NPs interaction’ mode. The CDV-CeO_2_ NPs potential to displace probes from the DNA and RNA structure was assessed by performing the displacement experiment. To this end, the solution of AO, and HO (5.00 × 10^−6^ M) was titrated with DNA and RNA (1.76 × 10^−4^ M). Thereafter, CDV-CeO_2_ NPs (2.44 × 10^−5^ to 2.59 × 10^−4^ g/mL) were transferred to the previous mixture. AO (noted from 640 to 730 nm by exciting at 502 nm), and HO (noted from, 350 to 650 nm by exciting at 340 nm).

## CONCLUSIONS

The use of Cydonia oblonga peel extract in the green synthesis process provided an environmentally friendly method for producing CeO_2_ NPs. These green-synthesized CeO_2_ NPs were then functionalized with the anti-DNA virus agent CDV. The CDV-CeO_2_ NPs were characterized using various methods. This research indicates that the CDV-CeO_2_ NPs show an inhibitory potential on cancerous cell line (MCF-7). Employing nucleic acids (DNA and RNA), the molecular recognition process and binding affinity of CDV-CeO_2_ NPs was deciphered by multi-spectroscopic methods. The evaluation of the fluorescence spectra and UV–visible absorption showed a strong association of the CDV-CeO_2_ NPs with RNA/DNA. Nonetheless, it is necessary to conduct a more thorough investigation to decipher the action mechanism before the assumption of a more significant role for the biosynthesized CDV-CeO_2_ NPs for medicinal purposes.
